# Supplementary material for: Cancer cell histone density links global histone acetylation, mitochondrial proteome and histone acetylase inhibitor sensitivity
Source: Commun Biol. 2022 Aug 27;5:882. doi: 10.1038/s42003-022-03846-3 (PMC9420116; doi:10.1038/s42003-022-03846-3)
Supplement: Supplementary file 2 — Supplementary Information [file 42003_2022_3846_MOESM2_ESM.pdf]

Cancer cell histone density links global histone acetylation, mitochondrial proteome and histone acetylase inhibitor sensitivity

Christopher Bruhn [1,2,*], Giulia Bastianello [1,3] and Marco Foiani [1,3,*]

[1] The FIRC Institute of Molecular Oncology (IFOM), Milan, Italy

[2] Present address: Evotec International GmbH, Göttingen, Germany

[3] Università degli Studi di Milano, Milan, Italy

* Correspondence: bruhndatascience@gmail.com (C.B.) or marco.foiani@ifom.eu (M.F.)

Supplementary Figures

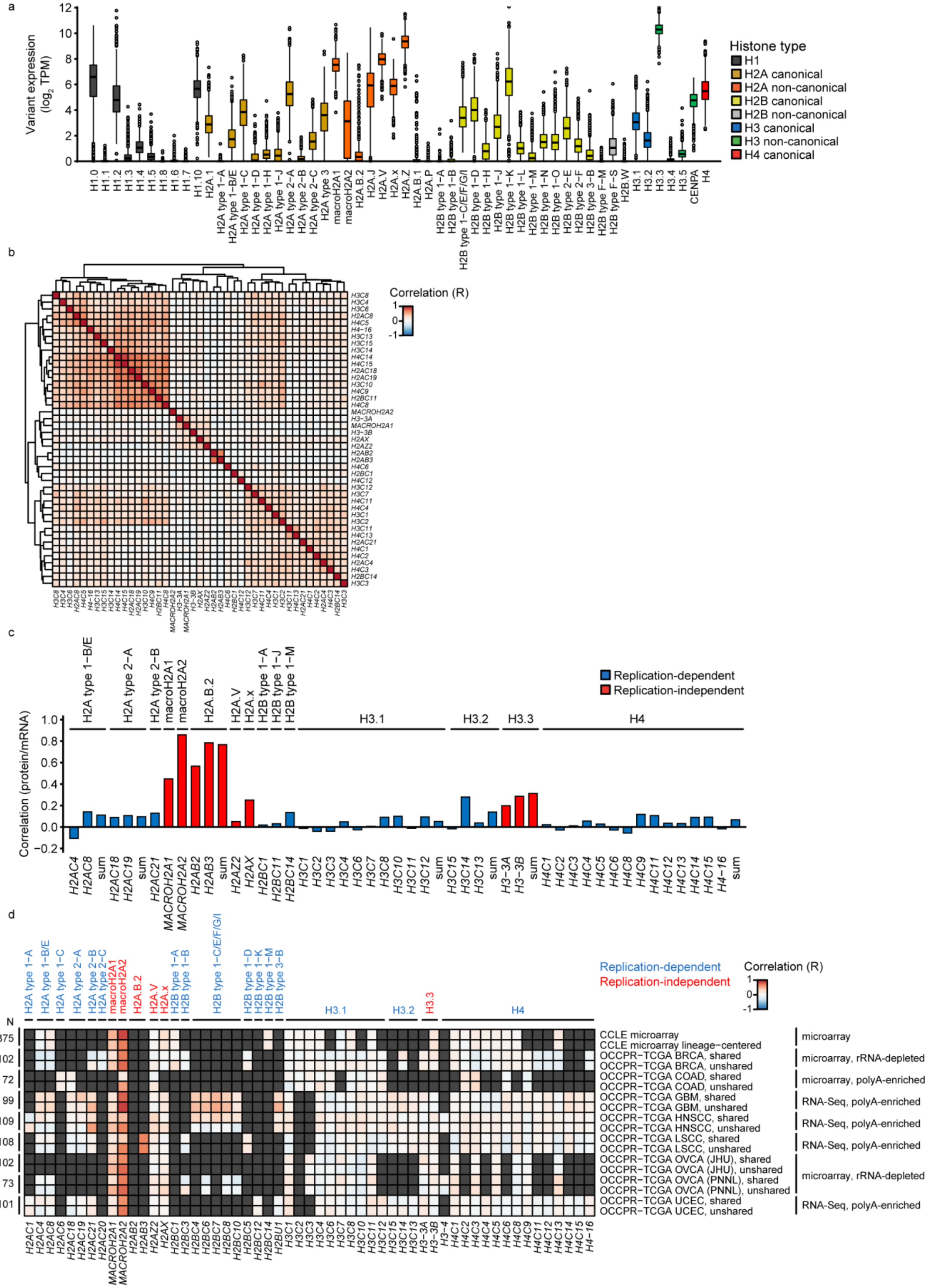
a
Variant expression (log_2 TPM)
Histone type
H1
H2A canonical
H2A non-canonical
H2B canonical
H2B non-canonical
H3 canonical
H3 non-canonical
H4 canonical
H1.0 H1.1 H1.2 H1.3 H1.4 H1.5 H1.6 H1.8 H1.7 H1.10 H2A.1 H2A type 1−A H2A type 1−B/E H2A type 1−C H2A type 1−D H2A type 1−H H2A type 1−J H2A type 2−A H2A type 2−B H2A type 2−C H2A type 3 macroH2A1 macroH2A2 H2A.B.2 H2A.J H2A.V H2A.x H2A.Z H2A.B.1 H2A.P H2B type 1−A H2B type 1−B H2B type 1−C/E/F/G/I H2B type 1−D H2B type 1−H H2B type 1−J H2B type 1−K H2B type 1−L H2B type 1−M H2B type 1−N H2B type 1−O H2B type 2−E H2B type 2−F H2B type 3−B H2B type F−M H2B type F−S H2B.W H3.1 H3.2 H3.3 H3.4 H3.5 CENPA H4
b
Correlation (R)
c
Correlation (protein/mRNA)
Replication-dependent
Replication-independent
H2A type 1−B/E
H2A type 2−A
H2A type 2−B
macroH2A1
macroH2A2
H2A.B.2
H2A.V
H2A.x
H2B type 1−A
H2B type 1−J
H2B type 1−M
H3.1
H3.2
H3.3
H4
d
Replication-dependent
Replication-independent
Correlation (R)
N
CCLE microarray
CCLE microarray lineage−centered
OCCPR−TCGA BRCA, shared
OCCPR−TCGA BRCA, unshared
OCCPR−TCGA COAD, shared
OCCPR−TCGA COAD, unshared
OCCPR−TCGA GBM, shared
OCCPR−TCGA GBM, unshared
OCCPR−TCGA HNSCC, shared
OCCPR−TCGA HNSCC, unshared
OCCPR−TCGA LSCC, shared
OCCPR−TCGA LSCC, unshared
OCCPR−TCGA OVCA (JHU), shared
OCCPR−TCGA OVCA (JHU), unshared
OCCPR−TCGA OVCA (PNNL), shared
OCCPR−TCGA OVCA (PNNL), unshared
OCCPR−TCGA UCEC, shared
OCCPR−TCGA UCEC, unshared
microarray
microarray, rRNA-depleted
microarray, polyA-enriched
RNA-Seq, polyA-enriched
RNA-Seq, polyA-enriched
RNA-Seq, polyA-enriched
microarray, rRNA-depleted
RNA-Seq, polyA-enriched
375
102
72
99
109
108
102
73
101

Supplementary Figure 1. Analysis of histone isoform expression.

a Histone mRNA levels from the CCLE expression (RNA-Seq) dataset, summarized by histone variant. The x axis shows the histone variants as named in the HISTome2 database. The y axis shows the estimated $\log_2$ histone variant expression, which was calculated as read sum of all genes encoding the respective variant. The boxplots represent the variant expression distribution across the cancer cell lines included in the CCLE dataset (N = 1305). The colors represent the histone type.

b Correlation of histone mRNAs across cancer cell lines. The heatmap represents Pearson correlation coefficients of histone mRNA levels from the CCLE RNA-Seq expression dataset, organized by hierarchical clustering. Cell lines covered by both RNA-Seq and proteomics datasets (N = 372) were used.

c Correlation of histone proteins vs. mRNAs across cancer cell lines (N = 372). The analysis and representation are equivalent to Fig. 1d, but data were not lineage-centered before correlation analysis.

d Correlation of histone mRNAs across cancer cell lines and patient samples. The heatmap represents Pearson correlation coefficients of histone protein variants (indicated above the heatmap) vs. the encoding mRNAs (indicated below the heatmap). Mean mRNA and protein expression levels per patient were calculated when more than one tumor sample was analyzed from the same patient. The font colors represent the histone classification by replication dependence. Shared and unshared indicate the peak area definition in the cited proteomics study. Study references are provided in the main text. The exact number of samples per study (N) is indicated.

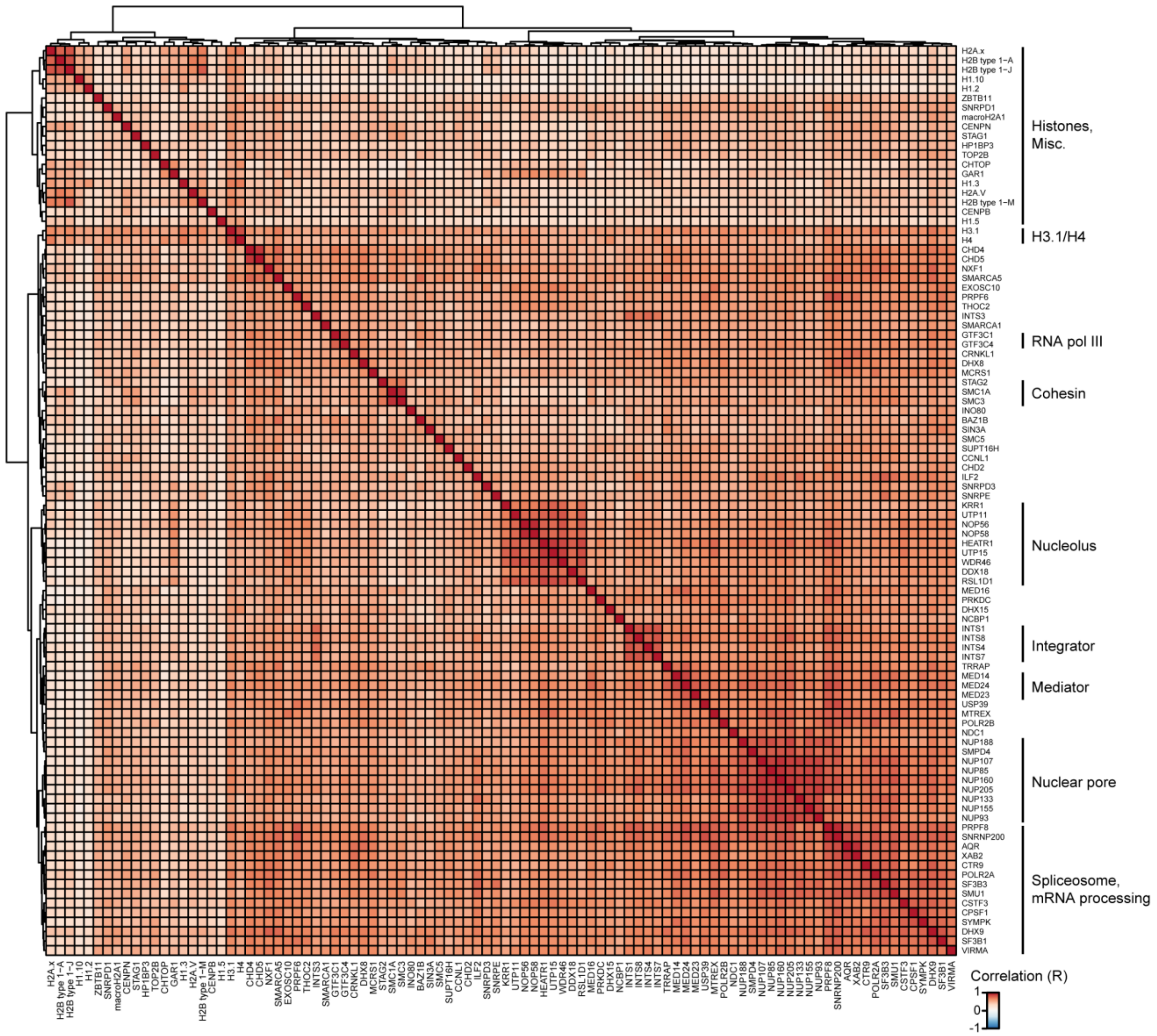
H2A.x
H2B type 1−A
H2B type 1−J
H1.10
H1.2
ZBTB11
SNRPD1
macroH2A1
CENPN
STAG1
HP1BP3
TOP2B
CHTOP
GAR1
H1.3
H2A.V
H2B type 1−M
CENPB
H1.5
H3.1
H4
CHD4
CHD5
NXF1
SMARCA5
EXOSC10
PRPF6
THOC2
INTS3
SMARCA1
GTF3C1
GTF3C4
CRNKL1
DHX8
MCRS1
STAG2
SMC1A
SMC3
INO80
BAZ1B
SIN3A
SMC5
SUPT16H
CCNL1
CHD2
ILF2
SNRPD3
SNRPE
KRR1
UTP11
NOP56
NOP58
HEATR1
UTP15
WDR46
DDX18
RSL1D1
MED16
PRKDC
DHX15
NCBP1
INTS1
INTS8
INTS4
INTS7
TRRAP
MED14
MED24
MED23
USP39
MTREX
POLR2B
NDC1
NUP188
SMPD4
NUP107
NUP85
NUP160
NUP205
NUP133
NUP155
NUP93
PRPF8
SNRNP200
AQR
XAB2
CTR9
POLR2A
SF3B3
SMU1
CSTF3
CPSF1
SYMPK
DHX9
SF3B1
VIRMA
Histones, Misc.
H3.1/H4
RNA pol III
Cohesin
Nucleolus
Integrator
Mediator
Nuclear pore
Spliceosome, mRNA processing
Correlation (R)
1
0
-1

Supplementary Figure 2. Correlation matrix of top histone correlating proteins.

Cell lines covered by both RNA-Seq and proteomics datasets (N = 373) were used. This Figure panel is an enlarged version of Fig. 2c with protein name labels.

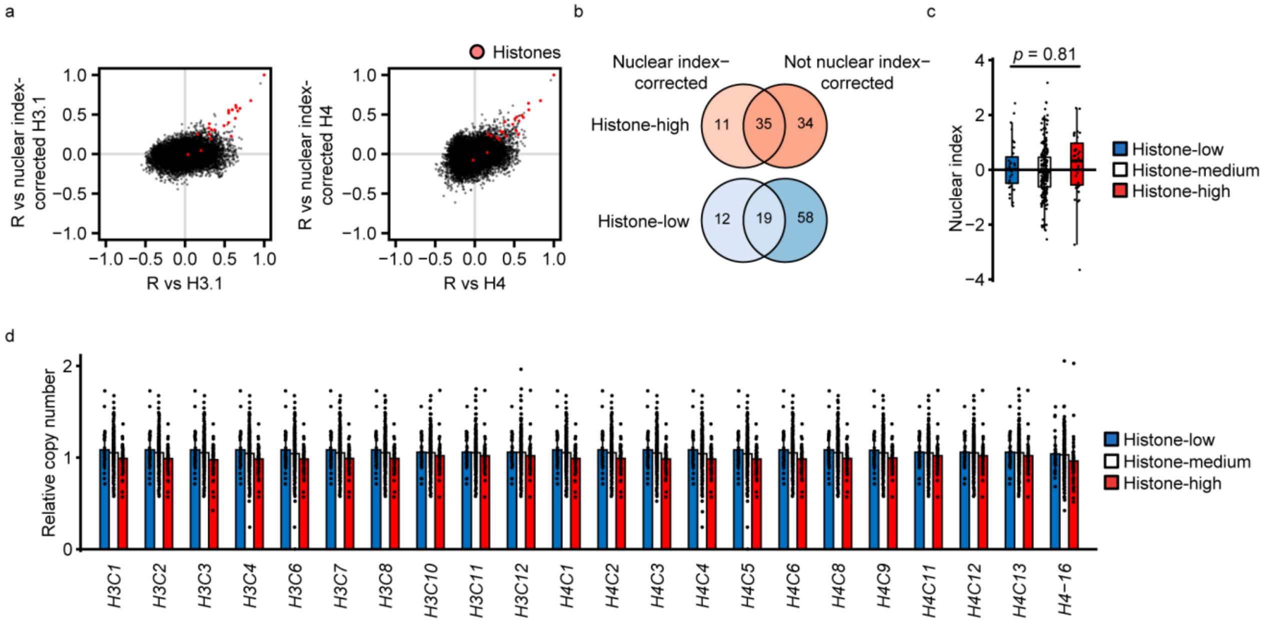

a
Histones
R vs nuclear index-corrected H3.1
R vs H3.1
R vs nuclear index-corrected H4
R vs H4
1.0
0.5
0.0
−0.5
−1.0
b
Nuclear index−corrected
Not nuclear index−corrected
Histone-high
11
35
34
Histone-low
12
19
58
c
p = 0.81
Nuclear index
Histone-low
Histone-medium
Histone-high
d
Relative copy number
Histone-low
Histone-medium
Histone-high
H3C1
H3C2
H3C3
H3C4
H3C6
H3C7
H3C8
H3C10
H3C11
H3C12
H4C1
H4C2
H4C3
H4C4
H4C5
H4C6
H4C8
H4C9
H4C11
H4C12
H4C13
H4−16

Supplementary Figure 3. Nuclear index, histone density and copy number variation.

a Effect of proteome-wide nuclear index correction on protein correlations with histones H3.1 and H4 across cancer cell lines (N = 373). The scatter plots show the Pearson correlation coefficients (R) of proteins vs. H3.1 and H4 with (y axis) and without (x axis) nuclear index correction of all protein levels. Each dot represents a protein covered by the CCLE proteome dataset. Histones are colored in red.

b The Venn diagram summarizes the number of cell lines classified as histone-high and -low out of 373 cell lines, with and without nuclear index correction of histone levels. A 20% increase or decrease, respectively, of both histones H3.1 and H4 was used as classification threshold.

c Nuclear index distribution in cell lines grouped by histone density (histone-high: 46, histone-low: 31, histone-medium: 296). The boxplots show nuclear index values of the indicated cell lines groups. Significance analysis was performed by two-tailed Student's *t*-test (two-sided, unpaired).

d Histone gene copy number variation in cell lines grouped by histone density and covered by CCLE CNV data (histone-high: 46, histone-low: 31, histone-medium: 295). The boxplots show relative mean gene copy numbers from the CCLE CNV dataset of genes encoding H3.1 and H4. The error bars represent the standard deviation.

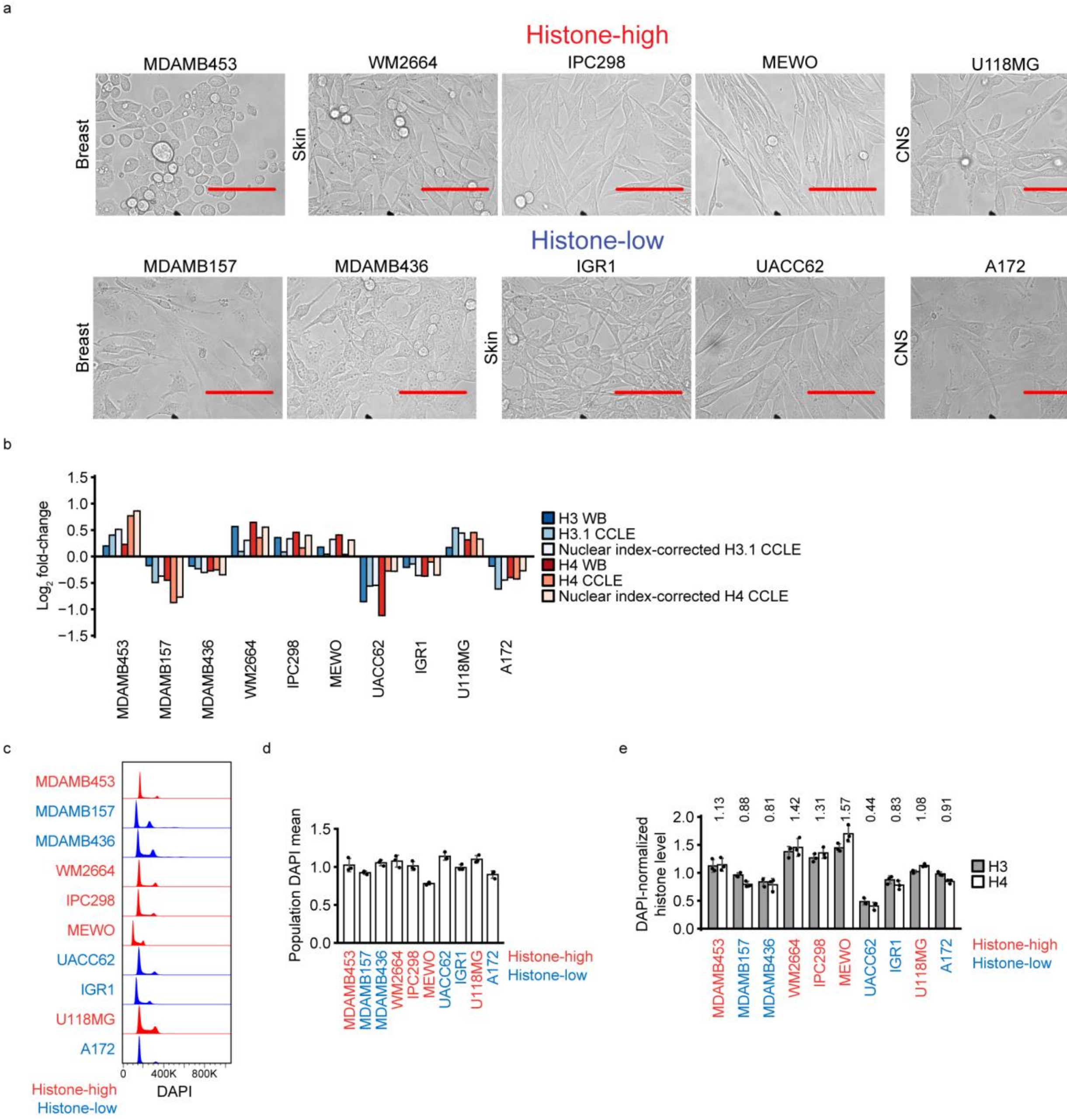
a
Histone-high
MDAMB453
WM2664
IPC298
MEWO
U118MG
Breast
Skin
CNS
Histone-low
MDAMB157
MDAMB436
IGR1
UACC62
A172
Breast
Skin
CNS
b
Log2 fold-change
1.5
1.0
0.5
0.0
−0.5
−1.0
−1.5
H3 WB
H3.1 CCLE
Nuclear index-corrected H3.1 CCLE
H4 WB
H4 CCLE
Nuclear index-corrected H4 CCLE
MDAMB453
MDAMB157
MDAMB436
WM2664
IPC298
MEWO
UACC62
IGR1
U118MG
A172
c
MDAMB453
MDAMB157
MDAMB436
WM2664
IPC298
MEWO
UACC62
IGR1
U118MG
A172
0
400K
800K
DAPI
Histone-high
Histone-low
d
Population DAPI mean
1.5
1.0
0.5
0.0
MDAMB453
MDAMB157
MDAMB436
WM2664
IPC298
MEWO
UACC62
IGR1
U118MG
A172
Histone-high
Histone-low
e
DAPI-normalized histone level
2.0
1.5
1.0
0.5
0.0
1.13
0.88
0.81
1.42
1.31
1.57
0.44
0.83
1.08
0.91
H3
H4
MDAMB453
MDAMB157
MDAMB436
WM2664
IPC298
MEWO
UACC62
IGR1
U118MG
A172
Histone-high
Histone-low

Supplementary Figure 4. Comparison of nuclear index-corrected and DNA-normalized histone density.

a Bright-field microscopy images of cancer cell lines. Cell morphology was regularly controlled during tissue culture and representative images are shown. The scale bars correspond to 100 µm.

b Qualitative comparison of experimental protein expression data with lineage-centered CCLE protein data, with and without nuclear index correction. Experimenal data are the log-transformed mean of histone expression from 3 parallel replicate cultures (N = 3) shown in Fig. 3h. CCLE predictions are based on a single sample (N = 1) per cell line.

c, d Flow cytometry analysis of DNA content. Three independent cultures per cell line were dissociated, subjected to ethanol fixation, stained with DAPI and analyzed on a flow cytometer with fixed voltage settings. Singulet gating was perfomed based on the side scatter parameter. The cell cycle distribution (**c**) and the mean DAPI intensity across the entire cell population as mean of 3 independent parallel replicate cultures (N = 3) ± standard deviation (**d**) are shown.

e Histone levels measured by Western blotting (Fig. 3h) normalized to mean DAPI intensities from **d**. Data are represented as mean of 3 independent replicate cultures ± standard deviation, with the lineage mean set to 1. The numbers above the bars represent the mean of H3 and H4 for the respective cell lines. The font color indicates the cell line classification from e by histone density. Significances were calculated by lineage with one-way ANOVA (breast: $p_{\text{ANOVA, H3}} = 0.015$, $p_{\text{ANOVA, H4}} = 6.4 \times 10^{-3}$; skin: $p_{\text{ANOVA, H3}} = 9.8 \times 10^{-8}$, $p_{\text{ANOVA, H4}} = 4.5 \times 10^{-7}$) with post hoc Tukey HSD test (breast: $p_{\text{MDAMB453 vs. MDAMB157, H3}} = 0.11$ $p_{\text{MDAMB453 vs. MDAMB157, H4}} = 0.011$, $p_{\text{MDAMB453 vs. MDAMB436, H3}} = 0.012$, $p_{\text{MDAMB453 vs. MDAMB436, H4}} = 0.010$; skin: $p_{\text{WM2664 vs. UACC62, H3}} = 3.6 \times 10^{-7}$, $p_{\text{WM2664 vs. UACC62, H4}} = 4.5 \times 10^{-6}$, $p_{\text{WM2664 vs. IGR1, H3}} = 7.1 \times 10^{-5}$, $p_{\text{WM2664 vs. IGR1, H4}} = 2.3 \times 10^{-4}$, $p_{\text{IPC298 vs. UACC62, H3}} = 1.3 \times 10^{-6}$, $p_{\text{IPC298 vs. UACC62, H4}} = 1.1 \times 10^{-5}$, $p_{\text{IPC298 vs. IGR1, H3}} = 5.7 \times 10^{-4}$, $p_{\text{IPC298 vs. IGR1, H4}} = 8.3 \times 10^{-4}$, $p_{\text{MEWO vs. UACC62, H3}} = 1.8 \times 10^{-7}$, $p_{\text{MEWO vs. UACC62, H4}} = 6.3 \times 10^{-7}$, $p_{\text{MEWO vs. IGR1, H3}} = 2.2 \times 10^{-5}$, $p_{\text{MEWO vs. IGR1, H4}} = 1.5 \times 10^{-5}$) or two-sided, unpaired Student's t test (CNS: $p_{\text{U118MG vs. A172, H3}} = 0.17$, $p_{\text{U118MG vs. A172, H4}} = 6.2 \times 10^{-4}$).

CNS: central nervous system

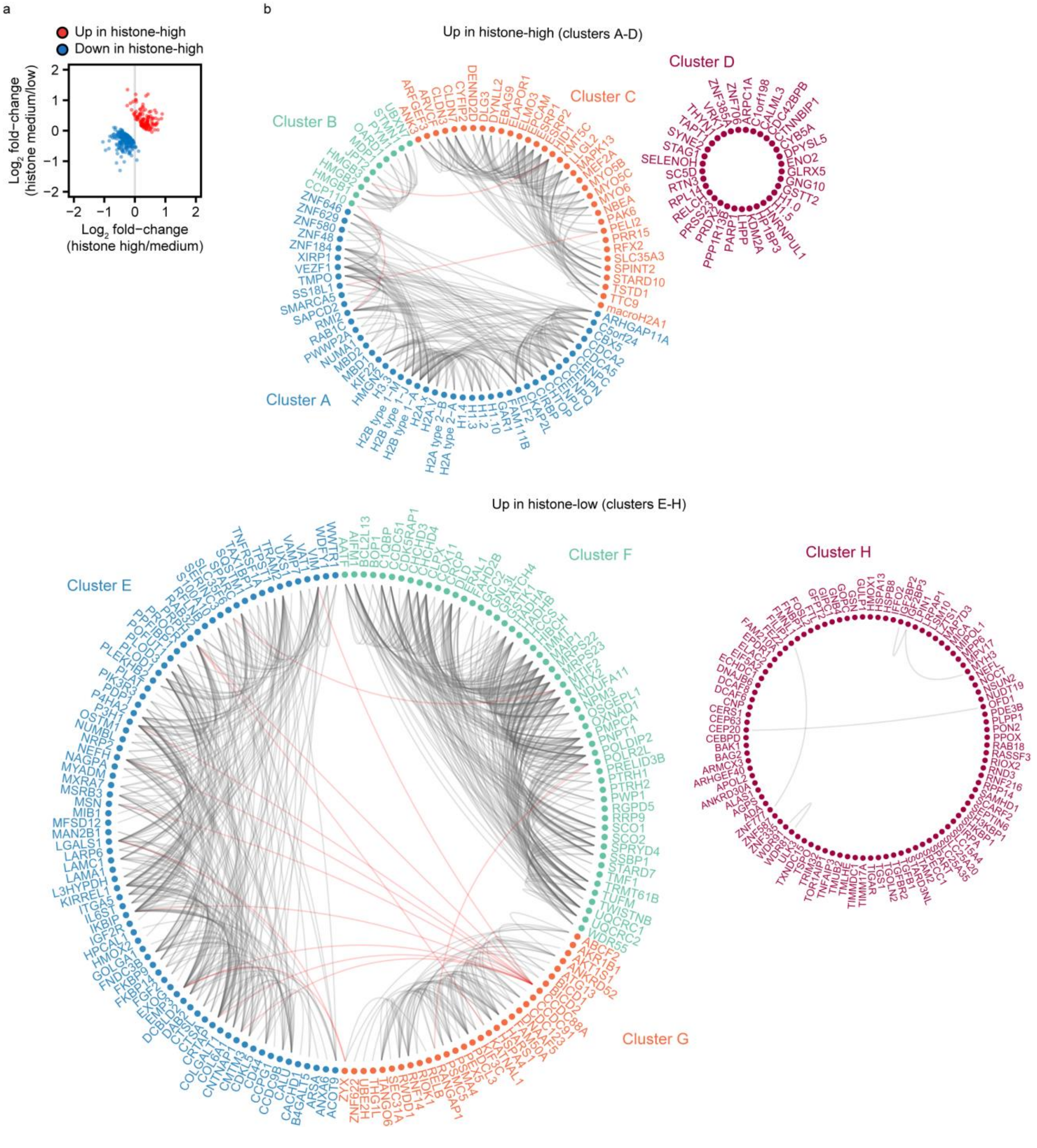
a
Up in histone-high
Down in histone-high
Log$_2$ fold−change (histone medium/low)
Log$_2$ fold−change (histone high/medium)
b
Up in histone-high (clusters A-D)
Cluster A
Cluster B
Cluster C
Cluster D
Up in histone-low (clusters E-H)
Cluster E
Cluster F
Cluster G
Cluster H

Supplementary Figure 5. The histone density-associated cancer cell line proteome.

a Fold-changes of differentially expressed proteins in histone-high (N = 37) vs. -low (N = 31) cells from Fig. 4a. The fold-changes vs. cells with normal histone content (N = 209) are represented. Each dot is a protein.

b Differentially expressed proteins in histone-high and -low cells. This Figure panel is a magnification of Fig. 4b.

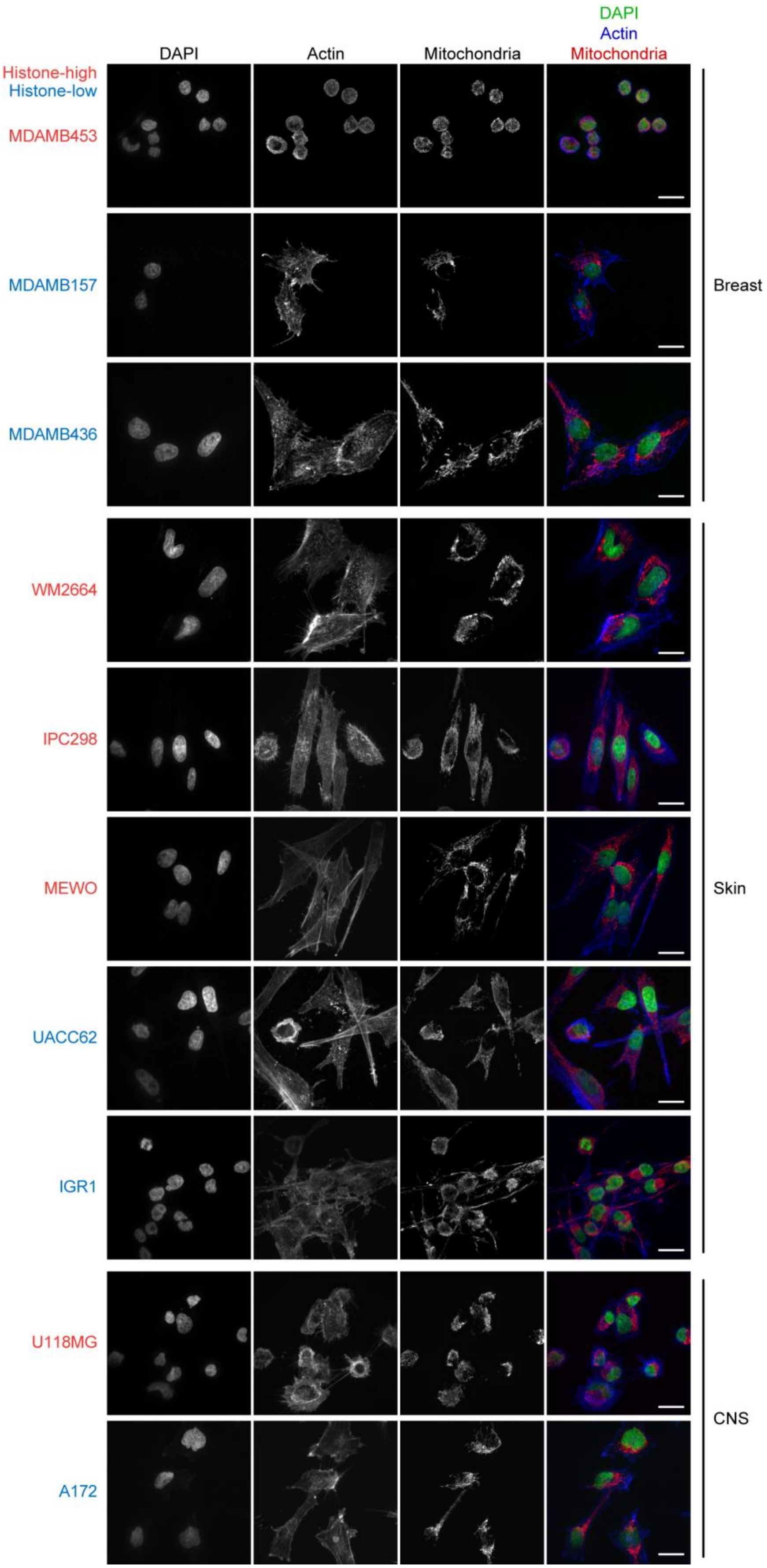
DAPI
Actin
Mitochondria
DAPI
Actin
Mitochondria
Histone-high
Histone-low
MDAMB453
MDAMB157
MDAMB436
WM2664
IPC298
MEWO
UACC62
IGR1
U118MG
A172
Breast
Skin
CNS

Supplementary Figure 6. Cell and mitochondrial morphology of experimental cell lines.

Projected confocal microscopy images of cancer cell lines. Cells were fixed, and mitochondria, actin cytoskeleton and nuclear DNA were labeled with an anti-mitochondria antibody, fluorophore-conjugated phalloidin and DAPI, respectively. 10 optical fields were acquired per cell line and representative images showing typcial cell morphology were selected. Intensities were adjusted for each cell line to allow visual comparison of the morphologies of subcellular structures. Scale bars correspond to 20 µm. CNS: central nervous system

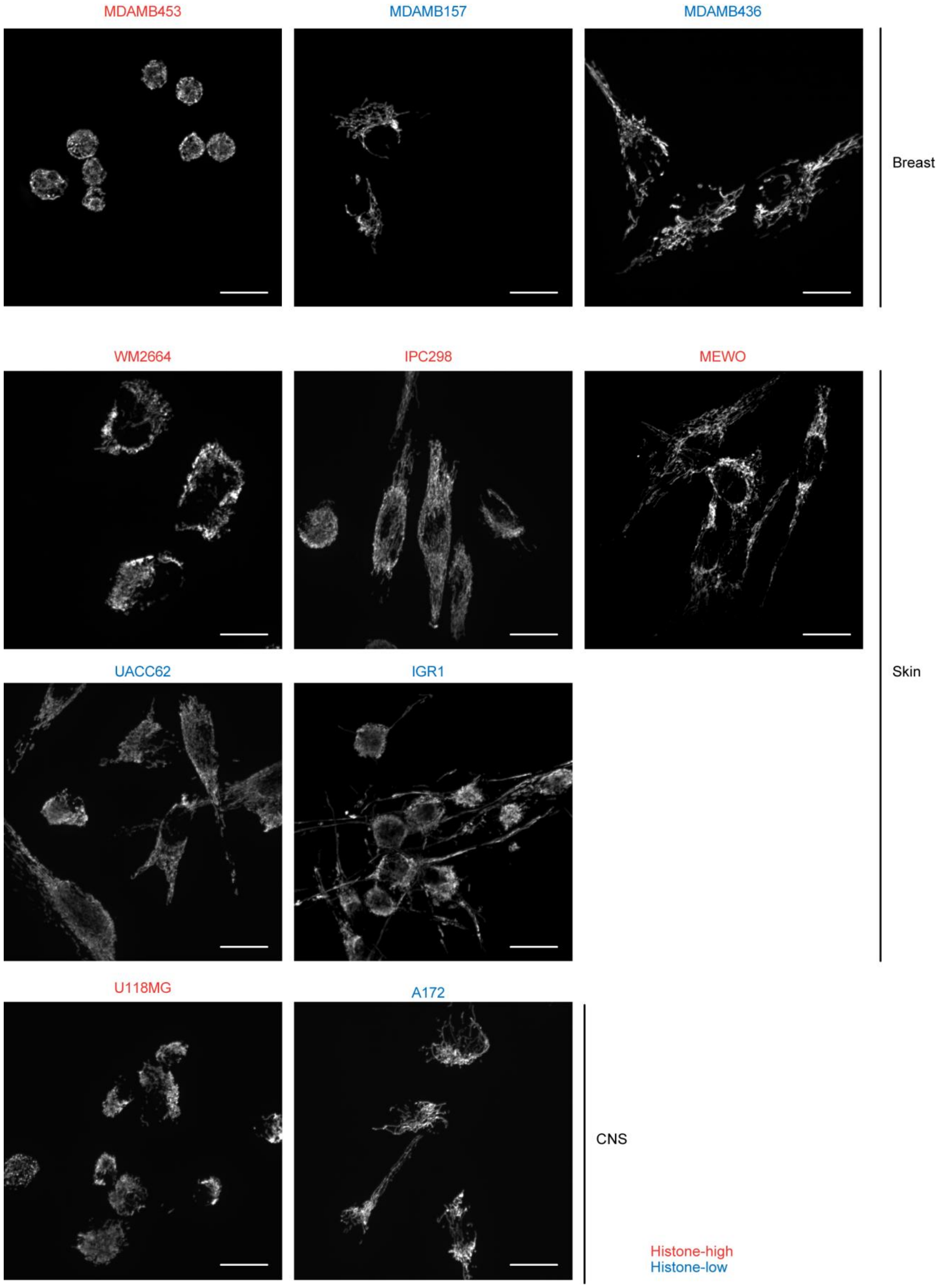
MDAMB453
MDAMB157
MDAMB436
Breast
WM2664
IPC298
MEWO
UACC62
IGR1
Skin
U118MG
A172
CNS
Histone-high
Histone-low

Supplementary Figure 7. Mitochondrial morphology of experimental cell lines.

Magnification of mitochondrial confocal microscopy images of cancer cell lines from Supplementary Fig. 6. Scale bars correspond to 20 µm. CNS: central nervous system

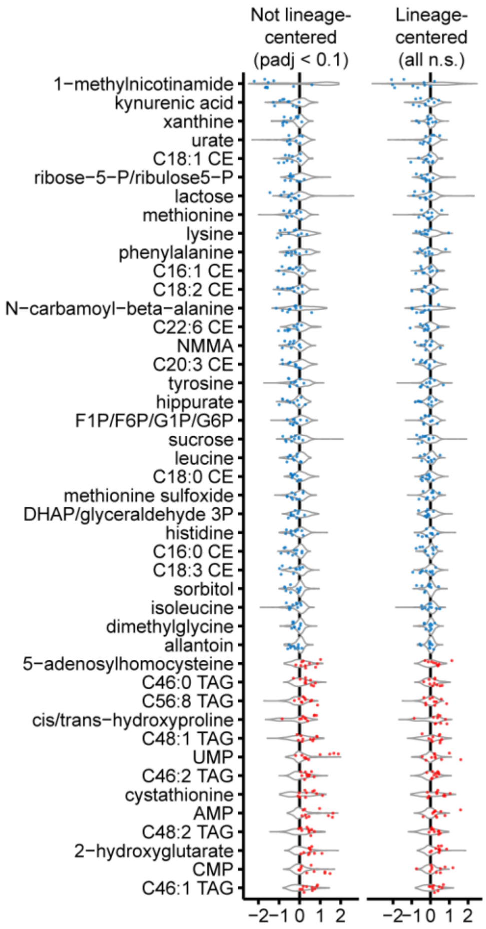
Not lineage-centered (padj < 0.1)
Lineage-centered (all n.s.)
1−methylnicotinamide
kynurenic acid
xanthine
urate
C18:1 CE
ribose−5−P/ribulose5−P
lactose
methionine
lysine
phenylalanine
C16:1 CE
C18:2 CE
N−carbamoyl−beta−alanine
C22:6 CE
NMMA
C20:3 CE
tyrosine
hippurate
F1P/F6P/G1P/G6P
sucrose
leucine
C18:0 CE
methionine sulfoxide
DHAP/glyceraldehyde 3P
histidine
C16:0 CE
C18:3 CE
sorbitol
isoleucine
dimethylglycine
allantoin
5−adenosylhomocysteine
C46:0 TAG
C56:8 TAG
cis/trans−hydroxyproline
C48:1 TAG
UMP
C46:2 TAG
cystathionine
AMP
C48:2 TAG
2−hydroxyglutarate
CMP
C46:1 TAG
−2 −1 0 1 2
−2 −1 0 1 2

Supplementary Figure 8. Metabolic characteristics of high histone density.

The CCLE metabolomics dataset was analyzed for significant differences between high confidence histone-high cell lines (Fig. 5c, N = 10) vs. cell lines with normal or low histone density (N = 240). The distribution of values in cell lines with normal or low histone density is indicated by violin plots. The distribution of values in high confidence histone-high cell lines is overlaid as blue dots (significant decrease) or red dots (significant increase). Significance between the two groups was analyzed with two-tailed Student's *t*-test with Benjamini-Hochberg correction ($p_{adj} < 0.1$, fold-change > 1.2). Only metabolites with significant differences in the comparison using non-lineage centered data are shown. Note that none of the metabolites are significant when using lineage-centered data.

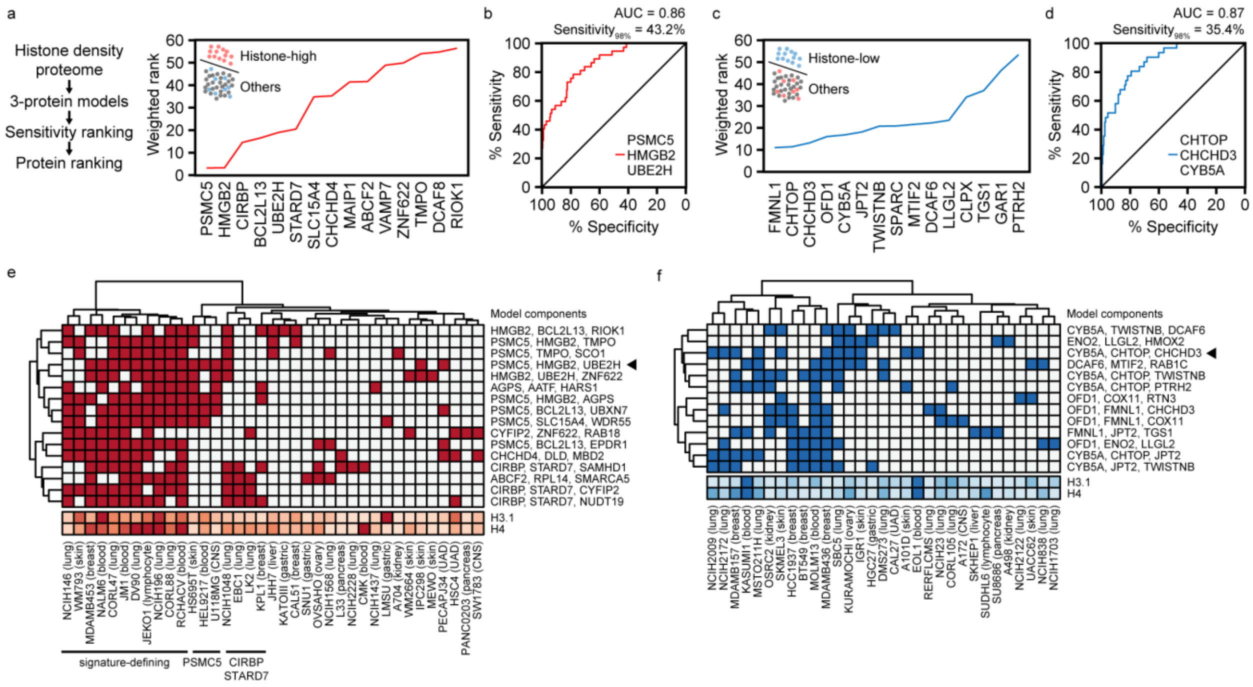

a
Histone density proteome
3-protein models
Sensitivity ranking
Protein ranking
Weighted rank
Histone-high
Others
PSMC5
HMGB2
CIRBP
BCL2L13
UBE2H
STARD7
SLC15A4
CHCHD4
MAIP1
ABCF2
VAMP7
ZNF622
TMPO
DCAF8
RIOK1
b
AUC = 0.86
Sensitivity98% = 43.2%
% Sensitivity
% Specificity
PSMC5
HMGB2
UBE2H
c
Weighted rank
Histone-low
Others
FMNL1
CHTOP
CHCHD3
OFD1
CYB5A
JPT2
TWISTNB
SPARC
MTIF2
DCAF6
LLGL2
CLPX
TGS1
GAR1
PTRH2
d
AUC = 0.87
Sensitivity98% = 35.4%
% Sensitivity
% Specificity
CHTOP
CHCHD3
CYB5A
e
Model components
HMGB2, BCL2L13, RIOK1
PSMC5, HMGB2, TMPO
PSMC5, TMPO, SCO1
PSMC5, HMGB2, UBE2H
HMGB2, UBE2H, ZNF622
AGPS, AATF, HARS1
PSMC5, HMGB2, AGPS
PSMC5, BCL2L13, UBXN7
PSMC5, SLC15A4, WDR55
CYFIP2, ZNF622, RAB18
PSMC5, BCL2L13, EPDR1
CHCHD4, DLD, MBD2
CIRBP, STARD7, SAMHD1
ABCF2, RPL14, SMARCA5
CIRBP, STARD7, CYFIP2
CIRBP, STARD7, NUDT19
H3.1
H4
signature-defining
PSMC5
CIRBP STARD7
f
Model components
CYB5A, TWISTNB, DCAF6
ENO2, LLGL2, HMOX2
CYB5A, CHTOP, CHCHD3
DCAF6, MTIF2, RAB1C
CYB5A, CHTOP, TWISTNB
CYB5A, CHTOP, PTRH2
OFD1, COX11, RTN3
OFD1, FMNL1, CHCHD3
OFD1, FMNL1, COX11
FMNL1, JPT2, TGS1
OFD1, ENO2, LLGL2
CYB5A, CHTOP, JPT2
CYB5A, JPT2, TWISTNB
H3.1
H4

Supplementary Figure 9. Protein-based prediction of histone density.

a, b Proteins of the histone density-associated proteome were ranked for their ability to correctly classify histone-high cells (N = 37) as histone-high in comparison with histone-low (N = 31) and -medium (N = 209) (**a**). Predictive logistic models of combinations of 3 proteins were trained and ranked by sensitivity in the receiver operating characteristic statistics. The receiver operating characteristic curve for the strongest model is shown in **b**. Proteins were then ranked by their contribution to the strongest models. The weighted rank of the highest scoring proteins is shown in **a**.

c, d Proteins of the histone density-associated proteome were ranked for their ability to correctly classify histone-low cells (N = 31) as histone-low vs. histone-high (N = 37) and -medium (N = 209) cells, in analogy to **a** and **b**.

e, f The top predictive models for all histone-high (**e**) or -low (**f**) cell lines are shown. A filled square in the heatmaps indicate that the respective cell line was correctly classified by the listed model. Cell lines and models are organized by hierarchical clustering. The nuclear index-corrected histone H3.1 and H4 densities are shown below the classification heatmap. The strongest models are indicated by filled arrowheads. Note that among the histone-high cell lines (**e**), a particularly high histone density is associated with the successful prediction by various different models. These cell lines are labeled signature-defining. Two other clusters contain cell lines that are preferentially predicted by models containing a specific protein, by which they are labeled (PSMC5, CIRBP/STARD7).

AUC: area under the receiver operating characteristic curve, CNS: central nervous system, UAD: upper aerodigestive tract

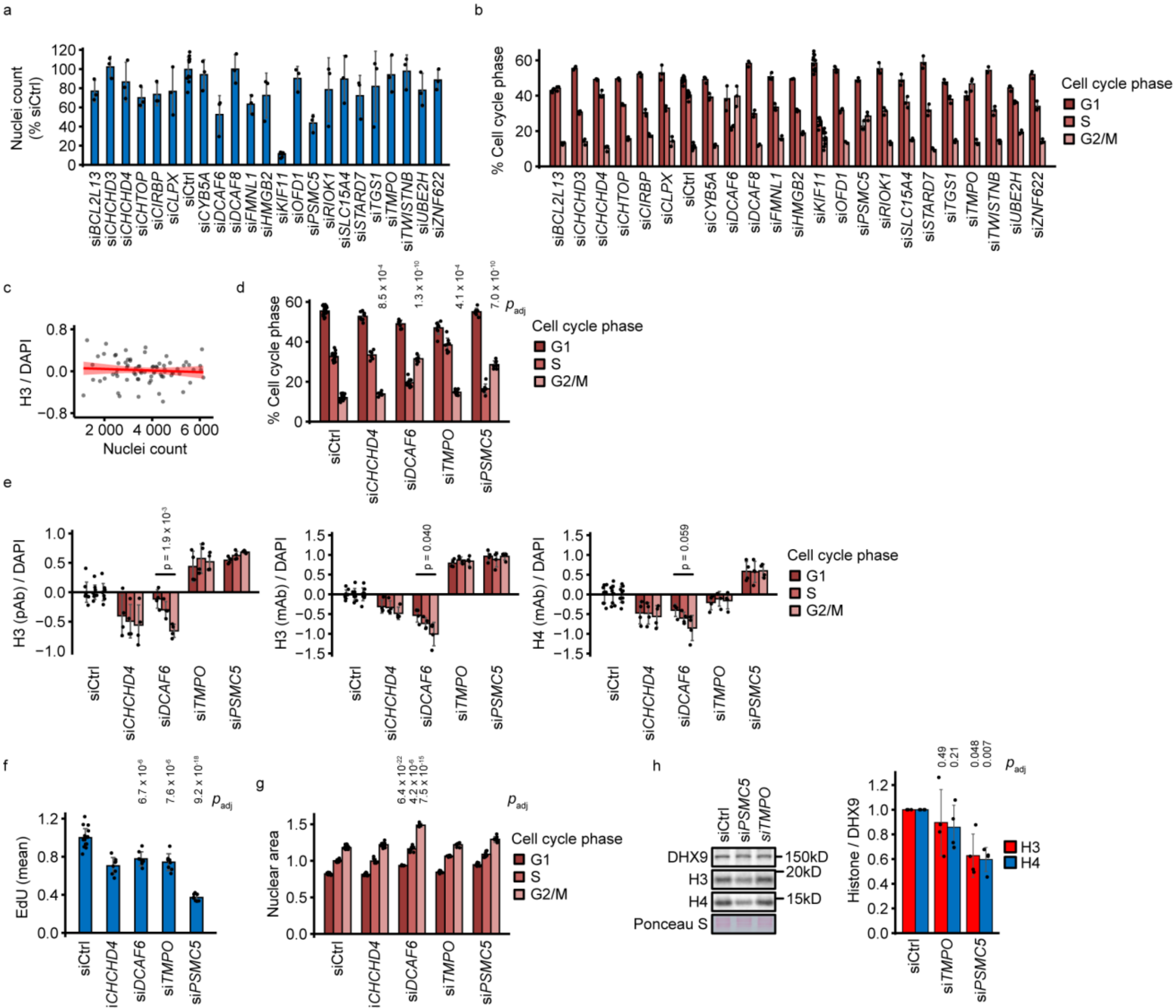
a
Nuclei count (% siCtrl)
siBCL2L13 siCHCHD3 siCHCHD4 siCHTOP siCIRBP siCLPX siCtrl siCYB5A siDCAF6 siDCAF8 siFMNL1 siHMGB2 siKIF11 siOFD1 siPSMC5 siRIOK1 siSLC15A4 siSTARD7 siTGS1 siTMPO siTWISTNB siUBE2H siZNF622
b
% Cell cycle phase
Cell cycle phase
G1
S
G2/M
c
H3 / DAPI
Nuclei count
d
% Cell cycle phase
8.5 x 10^-4
1.3 x 10^-10
4.1 x 10^-4
7.0 x 10^-10
p adj
siCtrl siCHCHD4 siDCAF6 siTMPO siPSMC5
e
H3 (pAb) / DAPI
p = 1.9 x 10^-3
H3 (mAb) / DAPI
p = 0.040
H4 (mAb) / DAPI
p = 0.059
f
EdU (mean)
6.7 x 10^-6
7.6 x 10^-6
9.2 x 10^-18
p adj
g
Nuclear area
6.4 x 10^-22
4.2 x 10^-6
7.5 x 10^-15
p adj
h
siCtrl siPSMC5 siTMPO
DHX9
H3
H4
Ponceau S
150kD
20kD
15kD
Histone / DHX9
0.49
0.21
0.048
0.007
p adj
H3
H4
siCtrl siTMPO siPSMC5

Supplementary Figure 10. High-content screening for modulators of histone density.

a, b Nucleus count (**a**) and cell cycle distribution (**b**) from the high-content imaging data presented in Fig. 6d. Three replicate plates (N = 3) with identical design were prepared, on which each siCtrl and si*KIF11* were transfected in 4 wells, and all other siRNAs were transfected in one well. Quantifications are based on at least 1123 cells per well (excluding si*KIF11*).

c Analysis of a potential impact of cell density vs. histone signal quantification in Fig. 6d. Each dot represents one screening well. The quantile regression of histone H3 signal / DAPI signal well mean vs. nuclei count shows that the nuclei count per well does not have a strong influence on the histone level readout.

d-g Cell cycle distribution (**d**), DAPI-normalized histone signal by cell cycle phase (**e**), nuclear EdU mean intensity of S phase cells (**f**) and nuclear area (**g**) from the high-content imaging data presented in Fig. 6f. Two separate experiments were performed for the validations using either polyclonal anti-H3 antibody or the combination of monoclonal H3 and H4 antibodies. In each experiment, cells were transfected with siRNAs in 9 (siCtrl), 3 (si*KIF11*) or 4 (all other siRNAs) replicate wells. Quantifications are based on at least 1123 and 1174 cells per well for the pAb H3 and the mAb H3/H4 screens, respectively (excluding si*KIF11*). Significance of G2 phase percentage (**d**), EdU incorporation (**f**) and nuclear area (**g**) against the scrambled contol siRNA was analyzed with two-tailed, unpaired Student‘s *t*-test with Benjamini-Hochberg correction. Significance of histone levels in G2 vs. G1 phase in si*DCAF6*-transfected cells (**e**) was analyzed with two-tailed, unpaired Student‘s *t*-test.

h Western blot validation of histone modulators. MDA-MB-231 cells were transfected with the indicated siRNAs and protein lysates were produced after 3 days. Protein levels were analyzed by Western blot. The barplot represents histone levels normalized to DHX9. Note that the Western blot data for the presented siRNAs do not validate the data from Fig. 6f. Significance against the scrambled contol siRNA was analyzed with two-tailed, unpaired Student‘s *t*-test with Benjamini-Hochberg correction (N = 4 independent siRNA transfections).

Data are represented as mean ± standard deviation. siCtrl is a scrambled siRNA.

Figure 3g

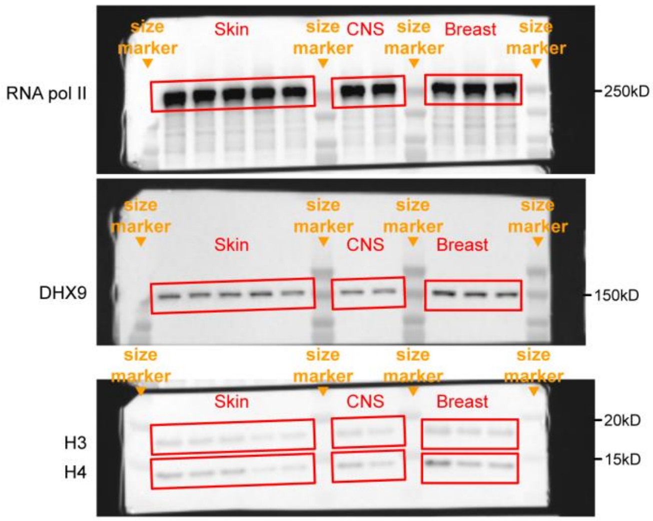

Supplementary Figure 10h, Figure 6g

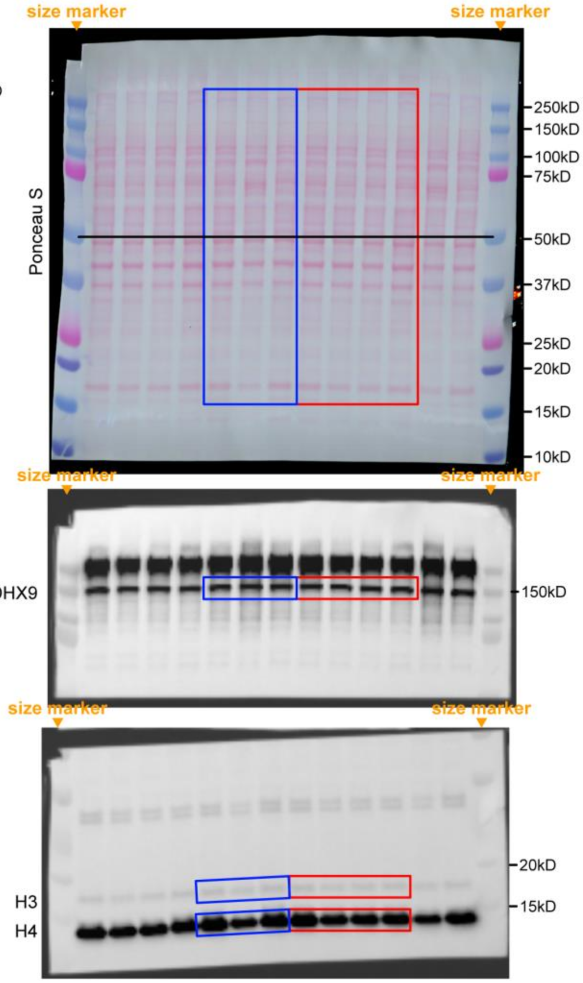

Figure 4d

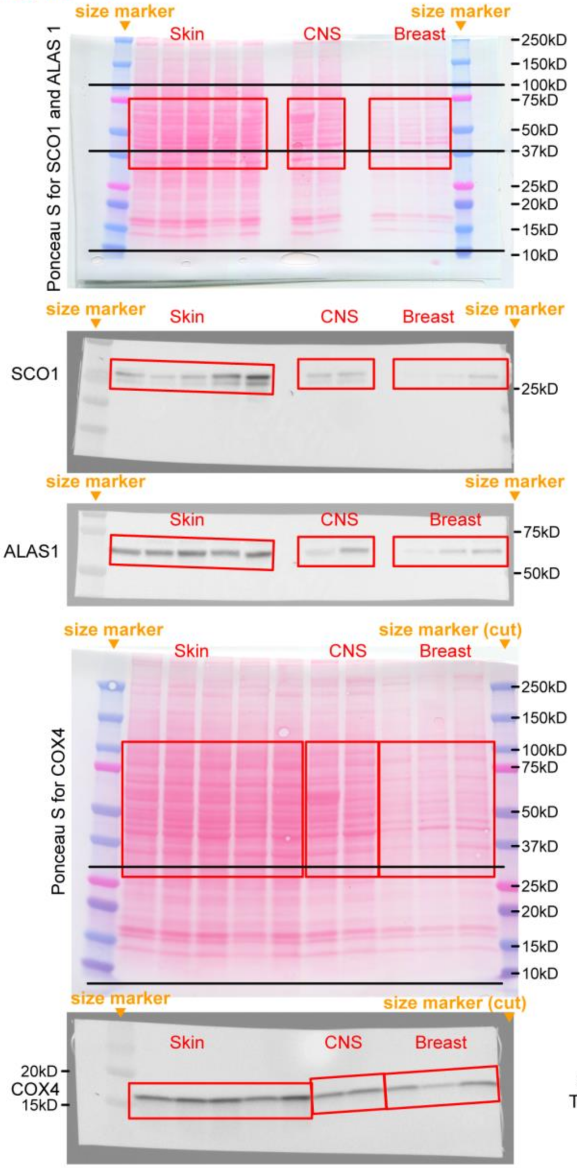

Figure 6h

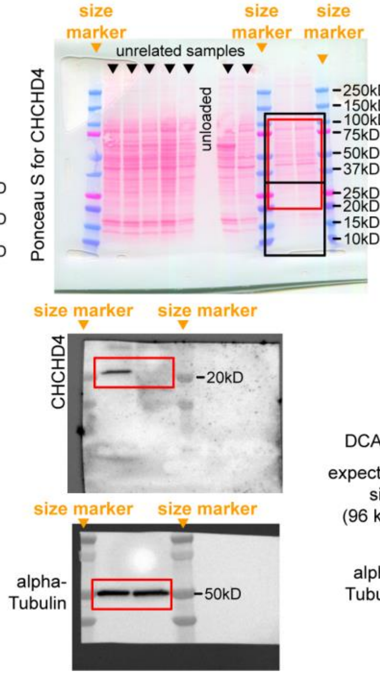

Figure 6i

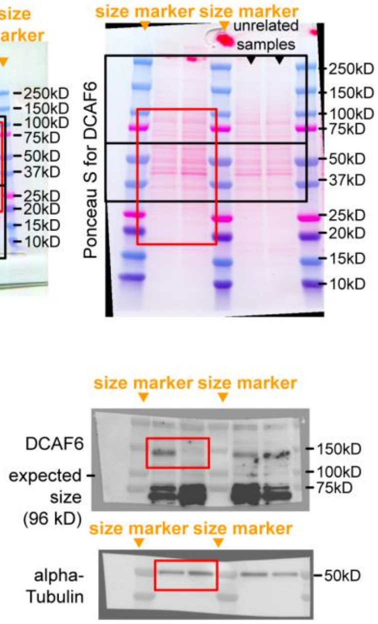

Supplementary Figure 11. Western blot scans.

Black boxes indicate membrane cuts. Red boxes indicate image cropping.

CNS: central nervous system

Figure 6b

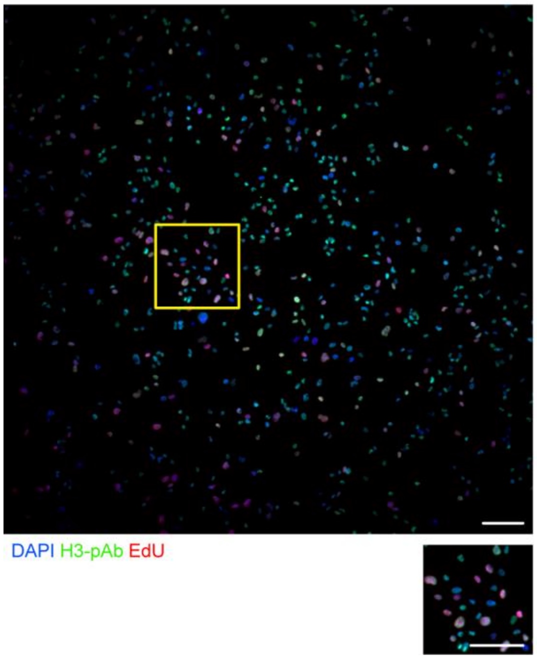

Figure 6e

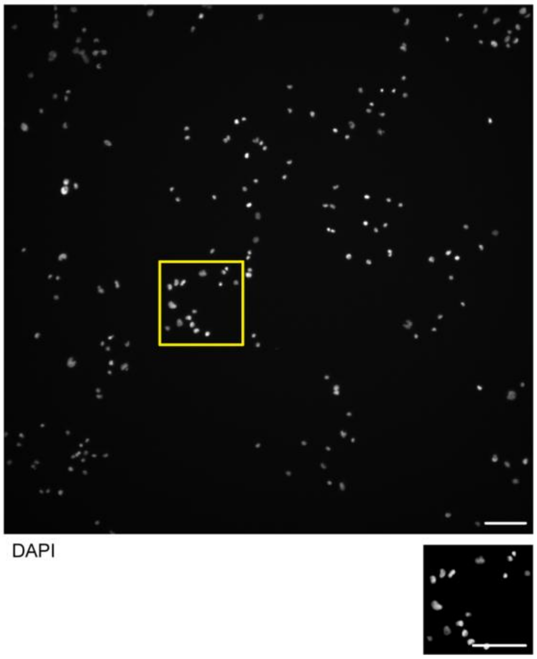

Supplementary Figure 12. Cropping of immunocytochemistry images.

The scale bars correspond to 100 µm.
