# Supplementary material for: Cancer cell histone density links global histone acetylation, mitochondrial proteome and histone acetylase inhibitor sensitivity
Source: Commun Biol. 2022 Aug 27;5:882. doi: 10.1038/s42003-022-03846-3 (PMC9420116; doi:10.1038/s42003-022-03846-3)
Supplement: Supplementary file 3 — Description of Additional Supplementary Files [file 42003_2022_3846_MOESM3_ESM.pdf]

Description of Additional Supplementary Files

File name: Supplementary Data 1
Description: Cell line classification by histone density.

File name: Supplementary Data 2
Description: Significance analysis of proteins in histone-high vs. -low cells.

File name: Supplementary Data 3
Description: Significance analysis of mRNAs in histone-high vs. -low cells.

File name: Supplementary Data 4
Description: Regulatory target gene set enrichment analysis on the mRNA fold-changes between histone-high and -low cells.

File name: Supplementary Data 5
Description: Significantly altered mRNAs representing targets of enriched transcription factor signatures in histone-high vs. histone-low cells.

File name: Supplementary Data 6
Description: Weighted protein ranks for the classification of histone-high cell lines.

File name: Supplementary Data 7
Description: Weighted protein ranks for the classification of histone-low cell lines.
