# Supplementary material for: Cancer cell histone density links global histone acetylation, mitochondrial proteome and histone acetylase inhibitor sensitivity
Source: Commun Biol. 2022 Aug 27;5:882. doi: 10.1038/s42003-022-03846-3 (PMC9420116; doi:10.1038/s42003-022-03846-3)
Supplement: Supplementary file 11 — Reporting Summary [file 42003_2022_3846_MOESM11_ESM.pdf]

nature portfolio

Corresponding author(s): Christopher Bruhn, Marco Foiani

Last updated by author(s): Aug 1, 2022

Reporting Summary

Nature Portfolio wishes to improve the reproducibility of the work that we publish. This form provides structure for consistency and transparency in reporting. For further information on Nature Portfolio policies, see our Editorial Policies and the Editorial Policy Checklist.

Statistics

For all statistical analyses, confirm that the following items are present in the figure legend, table legend, main text, or Methods section.

n/a	Confirmed	
☐	☒	The exact sample size (n) for each experimental group/condition, given as a discrete number and unit of measurement
☐	☒	A statement on whether measurements were taken from distinct samples or whether the same sample was measured repeatedly
☐	☒	The statistical test(s) used AND whether they are one- or two-sided<br>*Only common tests should be described solely by name; describe more complex techniques in the Methods section.*
☒	☐	A description of all covariates tested
☐	☒	A description of any assumptions or corrections, such as tests of normality and adjustment for multiple comparisons
☐	☒	A full description of the statistical parameters including central tendency (e.g. means) or other basic estimates (e.g. regression coefficient) AND variation (e.g. standard deviation) or associated estimates of uncertainty (e.g. confidence intervals)
☐	☒	For null hypothesis testing, the test statistic (e.g. F, t, r) with confidence intervals, effect sizes, degrees of freedom and P value noted<br>*Give P values as exact values whenever suitable.*
☒	☐	For Bayesian analysis, information on the choice of priors and Markov chain Monte Carlo settings
☒	☐	For hierarchical and complex designs, identification of the appropriate level for tests and full reporting of outcomes
☒	☐	Estimates of effect sizes (e.g. Cohen's d, Pearson's r), indicating how they were calculated

Our web collection on statistics for biologists contains articles on many of the points above.

Software and code

Policy information about availability of computer code

Data collection	All code was written in R language (version 4.0.2), managed in RStudio (version 1.0.153), and run in a Windows 7 (64 bit) operative system. All code is available through Mendeley Data: doi:10.17632/68pd82kgpg.1.
Data analysis	All code was written in R language (version 4.0.2), managed in RStudio (version 1.0.153), and run in a Windows 7 (64 bit) operative system. All code is available through Mendeley Data: doi:10.17632/68pd82kgpg.1.

For manuscripts utilizing custom algorithms or software that are central to the research but not yet described in published literature, software must be made available to editors and reviewers. We strongly encourage code deposition in a community repository (e.g. GitHub). See the Nature Portfolio guidelines for submitting code & software for further information.

Data

Policy information about availability of data

All manuscripts must include a data availability statement. This statement should provide the following information, where applicable:

- Accession codes, unique identifiers, or web links for publicly available datasets
- A description of any restrictions on data availability
- For clinical datasets or third party data, please ensure that the statement adheres to our policy

The manuscript contains a data availability statement with a link to the data collection in Mendeley data.

Human research participants

Policy information about studies involving human research participants and Sex and Gender in Research.

Reporting on sex and gender	NA
Population characteristics	NA
Recruitment	NA
Ethics oversight	NA

Note that full information on the approval of the study protocol must also be provided in the manuscript.

Field-specific reporting

Please select the one below that is the best fit for your research. If you are not sure, read the appropriate sections before making your selection.

☒ Life sciences ☐ Behavioural & social sciences ☐ Ecological, evolutionary & environmental sciences

For a reference copy of the document with all sections, see nature.com/documents/nr-reporting-summary-flat.pdf

Life sciences study design

All studies must disclose on these points even when the disclosure is negative.

Sample size	Descriptive statistics on histone expression in cancer cell lines and cancer patients were applied to all available samples without exclusion (Fig. 1a-d, Supplementary Fig. 1a-d). Nuclear index calculation was based on cell lines covered by CCLE proteomics and RNA-Seq datasets (N = 372) (Fig. 2a-h, Supplementary Fig. 2). Nuclear index corrections and cell line classification by histone density were applied to the cell lines covered by the CCLE proteomics dataset (N = 373) (Fig. 3a-f, Supplementary Fig. 3a, b). Histone density validation (Fig. 3g, h) and DNA content analysis (Supplementary Fig. 4c-e) were performed with parallel experimental replicate cultures (N = 3), and statistics were calculated separately by lineage and quantified histone. Mitochondrial protein expression validation was performed in 5 histone-high and 5 histone-low cell lines (Fig. 4d, e). Cell line groups by histone density contain 46 (histone-high), 31 (histone-low), and 296 (histone-medium) cell lines (Supplementary Fig. 3c, d). Cell line groups by histone density filtered for lineages that contain at least one histone-high and at least one histone-low cell line contain 37 (histone-high), 31 (histone-low), and 209 (histone-medium) cell lines These groups were used for most statistical analysis between histone density groups (Fig. 4a-c, f, g, Fig. 5a, b, Supplementary Fig. 5a, b, Supplementary Fig. 9a-f). A group of high confidence histone-high cell lines (Fig. 5c, N = 10) was used in multiple omics comparisons vs. a pool of histone-low and -medium cell lines (N = 240) (Fig. 5d-f, Supplementary Fig. 8). Representative imaging Figure panels without formal experimental repetition (Supplementary Fig. 4a, Supplementary Fig. 6, Supplementary Fig. 7) or from high-throughput screening (Fig. 6b, c, e) are shown as illustrations of cell identity and morphology. The primary high-content imaging screen was performed in 3 replicate wells split to 3 plates, with 4 control siRNAs per plate (Fig. 6d, Supplementary Fig. 10a-c). The secondary high-content imaging screens were performed in 4 replicate wells on the same plate, with 9 control siRNAs (Fig. 6f, Supplementary Fig. 10d-g). siRNA valdation experiments were performed with 4 experimental replicates for histone regulation (Fig. 6g, Supplementary Fig. 10h), and in three technical replicates for knock-down quantification (Fig. 6h-j).
Data exclusions	No data exclusions were performed. CCLE cell line inclusions in statistics are explained in the manuscript.
Replication	High content analysis data were obtained with 3 antibodies in total. Relevant hits were independently validated by Western blot analysis.
Randomization	Sample randomization and blinding were not performed. However, the study is largely based on published data and high-content imaging approach, which avoids data acquisition bias.
Blinding	Sample randomization and blinding were not performed. However, the study is largely based on published data and high-content imaging approach, which avoids data acquisition bias.

Reporting for specific materials, systems and methods

We require information from authors about some types of materials, experimental systems and methods used in many studies. Here, indicate whether each material, system or method listed is relevant to your study. If you are not sure if a list item applies to your research, read the appropriate section before selecting a response.

Materials & experimental systems

n/a	Involved in the study	
☐	☒	Antibodies
☐	☒	Eukaryotic cell lines
☒	☐	Palaeontology and archaeology
☒	☐	Animals and other organisms
☒	☐	Clinical data
☒	☐	Dual use research of concern

Methods

n/a	Involved in the study	
☒	☐	ChIP-seq
☒	☐	Flow cytometry
☒	☐	MRI-based neuroimaging

Antibodies

Antibodies used

The following antibodies were used for Western blotting: rabbit polyclonal anti-histone H3 (EpiCypher, Cat# 13-0001, 1:5000), rabbit monoclonal anti-histone H4 clone D2X4V (Cell Signaling Technology, Cat# 13919, 1:4000), rabbit polyclonal anti-DHX9 (Atlas Antibodies, Cat# HPA028050, 1:2000), mouse monoclonal anti-RNA polymerase II (Santa Cruz Biotechnology, Cat# sc-47701, 1:500), rabbit polyclonal anti-CHCHD4 (Novus biologicals, Cat# NBP2-76390, 1:1000), rabbit polyclonal anti-DCAF6 (Novus biologicals, Cat# NB100-56434, 1:1000), rabbit polyclonal anti-SCO1 (Atlas antibodies, Cat# HPA021579, 1:1000), mouse monoclonal anti-ALAS1 (Santa Cruz Biotechnology, Cat# sc-365153, 1:200), mouse monoclonal anti-COX4 (Cell Signaling Technology, Cat# 11967, 1:5000), mouse monoclonal anti-α-Tubulin (Merck, Cat# T5168, 1:1000), goat polyclonal anti-mouse IgG (H + L)-HRP Conjugate (Bio-Rad, Cat# 1706516, 1:20000), goat polyclonal anti-rabbit IgG (H + L)-HRP Conjugate (Bio-Rad, Cat# 1706515, 1:20000).
The following antibodies were used for immunocytochemistry: rabbit polyclonal anti-histone H3 (Abcam, Cat# ab1791, 1:200), mouse monoclonal anti-histone H3 clone 1B1B2 (Cell Signaling Technology, Cat# 14269, 1:200), rabbit monoclonal anti-histone H4 clone D2X4V (Cell Signaling Technology, Cat# 13919, 1:200), mouse monoclonal anti-mitochondria (Abcam, Cat# ab92824, 1:500), donkey polyclonal anti-mouse AlexaFluor-Cy3 (Jackson ImmunoResearch, Cat# AB_2340813, 1:400), donkey polyclonal anti-rabbit AlexaFluor-488 (Jackson ImmunoResearch, Cat# AB_2313584, 1:400).

Validation

- rabbit polyclonal anti-histone H3 (EpiCypher, Cat# 13-0001): Validated by our lab in PMID 32814778, expected MW in WB, expected nuclear signal and correlation with DNA content in ICC
- rabbit polyclonal anti-histone H3 (Abcam, Cat# ab1791): Validated in manufacturer's website, expected MW in WB, expected nuclear signal and correlation with DNA content in ICC
- mouse monoclonal anti-histone H3 clone 1B1B2 (Cell Signaling Technology, Cat# 14269): Validated in manufacturer's website, expected MW in WB, expected nuclear signal and correlation with DNA content in ICC
- rabbit monoclonal anti-histone H4 clone D2X4V (Cell Signaling Technology, Cat# 13919): Validated in manufacturer's website, expected MW in WB, expected nuclear signal and correlation with DNA content in ICC
- rabbit polyclonal anti-DHX9 (Atlas Antibodies, Cat# HPA028050): Validated in manufacturer's website, expected MW in WB
- mouse monoclonal anti-RNA polymerase II (Santa Cruz Biotechnology, Cat# sc-47701): Validated in manufacturer's website, expected MW in WB
- rabbit polyclonal anti-CHCHD4 (Novus biologicals, Cat# NBP2-76390, 1:1000): Validated in manufacturer's website, expected MW in WB, consistent with siRNA knock-down
- rabbit polyclonal anti-DCAF6 (Novus biologicals, Cat# NB100-56434, 1:1000): Apparent molecular weight (130 kD) different higher than expected; validated by comparison with literature showing consistent appearance in WB at ~ 130 kD with diverse antibodies detecting DCAF6: PMID 28758620 Figure S4G, PMID 22177699 Figure 1B, PMID 29608040 Figure 1A, consistent with siRNA knock-down which was confirmed by qPCR
- rabbit polyclonal anti-SCO1 (Atlas antibodies, Cat# HPA021579, 1:1000): Validated in manufacturer's website, expected MW in WB
- mouse monoclonal anti-COX4 (Cell Signaling Technology, Cat# 11967, 1:5000):): Validated in manufacturer's website, expected MW in WB
- mouse monoclonal anti-α-Tubulin (Merck, Cat# T5168, 1:1000): Validated in manufacturer's website, expected MW in WB
- mouse monoclonal anti-mitochondria (Abcam, Cat# ab92824, 1:500): Validated in manufacturer's website, typical mitochondrial morphology observed in confocal microscopy in 10 cell lines

Eukaryotic cell lines

Policy information about cell lines and Sex and Gender in Research

Cell line source(s)	Cell lines were obtained from ATTC (MDA-MB-157, MDA-MB-453, WM-266-4, A-172, U118MG), NCI (MDA-MB-231, UACC-62), CLS (MDA-MB-436), DSMZ (IPC-298, IGR-1), and ICLC (MEWO)
Authentication	Cell morphologies were tested visually for all cell lines. Cell line validation was performed by STR profiling (gene print 10 system, Promega). Bright-field images of all used cell lines are provided in Supplementary Fig. 4a.
Mycoplasma contamination	All cell lines were tested negative for mycoplasm contamination by PCR (PMID: 15361652) and colorimetric assay (mycoalert detection kit, Lonza).
Commonly misidentified lines (See ICLAC register)	None of the used cell lines is listed as misidentified cell line in the ICLAC register.
